# The Effects of Warfarin on the Pharmacokinetics of Senkyunolide I in a Rat Model of Biliary Drainage After Administration of Chuanxiong

**DOI:** 10.3389/fphar.2018.01461

**Published:** 2018-12-12

**Authors:** Haigang Li, Yu Jiang, Yang Wang, Huiying Lv, Haitang Xie, Guoping Yang, Chengxian Guo, Jing Tang, Tao Tang

**Affiliations:** ^1^Department of Pharmacy, Changsha Medical University, Changsha, China; ^2^Institute of Integrative Chinese Medicine, Xiangya Hospital, Central South University, Changsha, China; ^3^Department of Gerontology, Affiliated Hospital of T.C.M. of Xinjiang Medical University, Urumqi, China; ^4^Hunan Agricultural Product Processing Institute, Hunan Academy of Agricultural Sciences, Changsha, China; ^5^Anhui Provincial Centre for Drug Clinical Evaluation, The First Affiliated Hospital of Wannan Medical College, Wuhu, China; ^6^Center of Clinical Pharmacology, The Third Xiangya Hospital, Central South University, Changsha, China

**Keywords:** senkyunolide I, Chuanxiong, warfarin, pharmacokinetics, UPLC-MS/MS, biliary drainage

## Abstract

The aim of this study was to elucidate the effects of warfarin on senkyunolide I in a rat model of biliary drainage after oral administration Chuanxiong extract based on pharmacokinetics. Thirty-two rats were randomly divided into four groups: CN, healthy rats after a single administration of Chuanxiong; CO, rats with biliary drainage after a single administration of Chuanxiong; WCN, healthy rats after the administration of Chuanxiong and warfarin; WCO, rats with biliary drainage after the administration of Chuanxiong and warfarin. A series of blood samples were collected at different time points before and after oral administration. An ultra-performance liquid chromatography coupled to tandem mass spectrometry (UPLC-MS/MS) method for quantification of the main components of Chuanxiong and methyclothiazide (internal standard) have been established. The validated method was successfully applied to a comparative pharmacokinetics study. After calculated by the DAS 2.1.1 software, the pharmacokinetics parameters of senkyunolide I showed a significant difference between the CN and CO groups, the AUC_0−t_, and C_max_ of CO group increased by 5.45, 4.02 folds, respectively. There was a significant difference between the WCO and WCN groups, the T_max_ of WCO group prolonged 67%; compared to the CN group, the AUC_0−t_, and C_max_ of WCN group raised 4.84, 3.49 folds, respectively; the T_max_ and C_max_ between the CO and WCO groups also showed a significant difference. The drug warfarin significantly affected the senkyunolide I disposition, which partly due to its enterohepatic circulation process in rat plasma after oral administration of Chuanxiong. The present study highlights an urgent evidence for drug-herb interactions.

## Introduction

There is widespread unrevealed use of herbal medicinal product (HMP) herbal medicine among Western doctors. Previous studies have indicated that the rate of HMP use is 75.4% in Singapore and more than 90.0% in Italy (Lim et al., 2005; Cuzzolin et al., 2007). The use of traditional Chinese medicine (TCM) is also common in various countries and regions, with an estimated 23.5% of patients in Italy and 26.0% in Hong Kong, including co-ingesting Chinese herbs and warfarin (Wong et al., 2003; Cuzzolin et al., 2007).

Rhizoma Chuanxiong (rhizomes of *Ligusticum chuanxiong* Hort.) is a commonly prescribed Chinese herbal medicine, and also known as Chuanxiong in China. Chuanxiong is used to relieve the pain induced by blood stagnation and has been used in China for centuries. According to TCM theory, the Chuanxiong pharmacological action is “to active blood circulation and relieve blood stasis” (Chinese Pharmacopoeia Committee, 2015). As an promoting blood circulation herbs, Chuanxiong is frequently used in the classical formulas, such as Xue Fu Zhu Yu decoction (Xing et al., 2016) and Bu Yang Huan Wu decoction (Cui et al., 2015), for cardiovascular disease treatment.

In some regions (such as Hong Kong) and China, warfarin often co-administrates with herbal prescriptions including Chuanxiong, and these integrative Chinese medicine methods are advised most widely for prevention of cardiovascular diseases in clinical practice (Feng et al., 2015; Zhang et al., 2016). Warfarin is the most widely used oral anticoagulant and is prescribed for patients to treat blood clots such as chronic atrial fibrillation, deep vein thrombosis, mechanical valves, and recurrent stroke (Piatkov et al., 2010). Annual prescriptions of warfarin are typically equal 0.5–1.5% of the population (Johnson et al., 2011). After the subject stops taking warfarin, it can exert anticoagulant effects for several days, while the novel oral anticoagulants (NOACs), such as apixaban, dabigatran, edoxaban, and rivaroxaban, do not have this unique benefit (Mega and Simon, 2015).

The Chinese herbal medicine Chuanxiong contains a hundred or more components according to the related materials. We have verified that Chuanxiong may significantly alter the pharmacokinetics of warfarin in rats, partly due to enterohepatic circulation (Li et al., 2016b). As a unique quality control bioactive component of Chuanxiong in Chinese Pharmacopoeia Committee (2015), ferulic acid cannot produce the similar effects of Chuanxiong on the pharmacokinetics of warfarin (Li et al., 2016a,b). We want to investigate that the effects of warfarin on the pharmacokinetics of Chuanxiong. Senkyunolide I (SEI), the main active ingredient of Chuanxiong, is used for treatment antimigraine and antioxidative damage (He et al., 2012; Wang et al., 2012), subordinate to “to active blood circulation and relieve blood stasis” of Chuanxiong according to the theory of TCM. Some published literatures indicated that SEI can be excreted mostly in rat bile after oral administration 2 h (Ma et al., 2015). The aim of this study was to elucidate the effects of warfarin on senkyunolide I in a rat model of biliary drainage after oral administration Chuanxiong extract based on pharmacokinetics.

## Materials and Methods

### Preparation of Chuanxiong Extract

Raw dried Rhizoma Chuanxiong was provided by the pharmacy of Xiangya Hospital, Central South University, Changsha, China. The specimen (voucher specimen no. 20140512, Chengdu, China) identified by Professor Suiyu Hu (Institute of Integrative Chinese Medicine, Xiangya Hospital, Central South University, Changsha, China). It was stored in the Laboratory of Ethnopharmacology of Xiangya Hospital, Central South University. Soaking in cold water for 30 min first, 100 g raw Chuanxiong Rhizoma thoroughly decocted in water (1:8, g/mL) for 1 h, and then the gruffs decocted an hour with water (1:6, g/mL) again, filtered through fourfolds gauze. The two filtrates were mixed and evaporated by rotary evaporation under vacuum at 60°C, until concentrated to 100 mL with rotary evaporation at 50°C, then the final decoction was deposited in 4°C refrigerator until use.

### Chemicals and Reagents

Senkyunolide I (C_12_H_16_O_4_, CAS, 94596-28-8, purity ≥ 98%; Lot, PA0804FA13), tetramethylpyrazine (C_8_H_12_N_2_, CAS, 1124-11-4, purity ≥ 98%; Lot, MUST-13022802), and levistilide A (C_24_H_28_O_4_, CAS, 88182-33-6, purity ≥ 98%; Lot, MUST-13020211) were purchased from Chengdu Must Bio-technology Co., Ltd. (Chengdu, China). Ferulic acid (C_10_H_10_O_4_, CAS, 1135-24-6, purity ≥ 99%; Lot, F1205050) was bought from Sigma-Aldrich (Netherlands). Warfarin sodium (C_19_H_15_NaO_4_, CAS, 129-06-6, purity ≥ 92.3%; Lot, 101163-201001) and methyclothiazide (C_9_H_11_Cl_2_N_3_O_4_S_2_, CAS, 135-07-9, Lot, 101163-201101; purity ≥ 99.6%; internal standard, IS) were obtained from the National Institutes for Food and Drug Control (Beijing, China). Formic acid was purchased from Tianjing Gangfu Fine Chemical Reagent Factory (Tianjing, China). HPLC grade methanol and acetonitrile were ordered from Merck (Darmstadt, Germany). Deionized water was clarified by a TKA Samrt2pure water purification system (German) with a sensitivity of 18.2 MO.

### Surgical Procedures

All animal procedures were approved by the Animal Ethics Committee of Central South University and were performed in strict compliance with our institutional protocols. Thirty-two specific pathogen free (SPF) grade Sprague-Dawley male rats (250–300 g), were obtained from Shanghai Laboratory Animal Center (SLAC). They were housed at 22–26°C and 45–75% relative humidity room in an SPF environment on 12:12 h day and night cycle with lights. All rats were fed standard rodent chow and got tap water *ad libitum*, except for the 12-h fast before the pharmacokinetics study. The experimental rats were divided into four groups at random, eight rats each group. CN Group: healthy rats after a single gastric-administration of 10 g/kg Chuanxiong; CO Group: rats subjected to biliary drainage after the gastric-administration of 10 g/kg Chuanxiong; WCN Group: healthy rats after the gastric-administration of Chuanxiong and warfarin sodium at doses of 10 g/kg and 0.5 mg/kg, respectively; and WCO Group: rats subjected to biliary drainage after the gastric-administration of Chuanxiong and warfarin sodium at doses of 10 g/kg and 0.5 mg/kg, respectively. In this study the dose of drugs calculated on the basis of the conventional dosage of clinical.

All rats were fasted for food but not water all-night before the experiments. After anesthetized with 0.4 mL 10% chloral hydrate per 100 g body weight by intraperitoneal injection, with an infrared lamp to maintain normal body temperature, bile drainage surgery was carried out under sterile conditions. After skin preparation, cuts the midline on the abdomen, opens up the liver, and notes the common bile duct of rat over the margin of duodenal bulb. Ligates the distal end of the bile duct and insert a polyethylene catheter (outer diameter is 1.2 mm and inner diameter 0.8 m) into the proximal part of common bile duct. After insertion, yellow bile could be seen either immediately or a few minutes, the free end of external catheter fixes to the peritoneum through a small hole made on the back of the rat’s neck and brought out of the body.

### Sample Collection and Preparation

On the second day after biliary drainage operation, blood samples were collected into heparinized tubes prior to treatment and the other different time points at 0.083, 0.25, 0.5, 1, 2, 4, 6, 8, 12, and 24 h post-dose. Centrifuged for 15 min at 3,000 rpm, then the supernatants were stored at −80°C until analysis. An aliquot of 100 μL of rat plasma was added with 10 μL of IS solution, 50 μL of 20% formic acid and 1 mL of methanol, which was followed by vortex-mixing for 3 min and hyperacoustic mixing for 5 min. Subsequently, the samples were centrifuged for 10 min at 3,000 rpm. Under a gentle stream of nitrogen the supernatants were evaporated at room temperature to dryness, followed by redissolution with 100 μL mixture of acetonitrile and water (50:50). This was followed by vortex-mixing for 3 min and hyperacoustic mixing for 5 min. The samples were then centrifuged for 10 min at 15,000 rpm, the supernatants were filtered through the 0.22-μm membrane, and 2 μL of the processed sample was injected into the autosampler for the analysis.

### Instrumentation and Analytical Conditions

The sample analysis was operated on an UPLC-MS/MS system. The Acquity^TM^ UPLC system (Waters Corporation, Milford, MA, United States) was composed of a column oven (set at 35°C), a binary solvent delivery manager and an autosampler (set at 10°C). Chromatographic separation was achieved on a Waters Acquity BEH C_18_ column (2.1 mm × 100 mm I.D., 1.7 μm, Waters, Wexford, Ireland). A mobile phase consisting of A (aqueous buffer containing 0.1% formic acid) and B (acetonitrile) was pumped at 0.3 mL/min. The following linear gradient elution was applied to the analyte: 0–0.25 min, 10% B; 0.25–5.0 min, 10–95% B; 5.0–8.0 min, holding at 95% B; then, between 8.0 and 8.2 min, an immediate decrease to 10% B (the initial conditions) for equilibration of the column. The typical injection volume was 2 μL. The detection system, a tandem quadrupole mass spectrometer (Waters Corporation, Manchester, United Kingdom), was performed using an electrospray ionization with the capillary voltage set at 2.5 KV, the desolvation temperature was fixed at 365°C, and the source temperature was set at 110°C in negative ion mode. Nitrogen was used for the cone gas flow (50 L/h) and desolvation gas flow (650 L/h). Argon was used as the collision gas at a flow rate of 0.2 mL/min for collision-induced dissociation. The Masslynx 4.1 software program (Waters Corporation) was used for data acquisition and processing. The multiple-reaction monitoring (MRM) mode was selected for quantitation the main bioactive components of Chuanxiong and IS, the precursors of which for the production ion transitions were as follows: tetramethylpyrazine, 137.10- 96.04; levistilide A, 381.09→190.92; SEI, 225.11→207.04; ferulic acid, 192.98→133.96; IS, 357.81→321.89. The data were gathered and analyzed using the DAS 2.1.1 software.

### Method Validation

The method was affirmed in terms of selectivity, linearity, accuracy, precision, matrix effects, extraction recovery, and stability according to FDA guidelines for bioanalytical method validation. Calibration curve samples were corrected by spiking blank plasma. To evaluate duplicate calibration standards, linearity of main bioactive components of Chuanxiong (ferulic acid, levistilide A, SEI, and tetramethylpyrazine) at six concentrations of blank plasma 5, 20, 100, 500, 2000, and 5000 ng/mL levels were dispensed and analyzed. The linearity of the method was calculated by analysis of standard plots associated with a six-point calibration curve. The overall inter- and intra-day variations all used five analytes.

### Statistical Analysis

Statistical evaluation was analyzed by SAS software (Cary, North Carolina). Data are expressed as the means with standard deviation (SD). The differences between the quantitative groups with normal distribution were evaluated with the Student’s *t*-test. The Wilcoxon signed rank test was used for abnormally distributed variables. Differences were considered statistically significant for *P* < 0.05.

## Results

### Specificity

**FIGURE 1:**
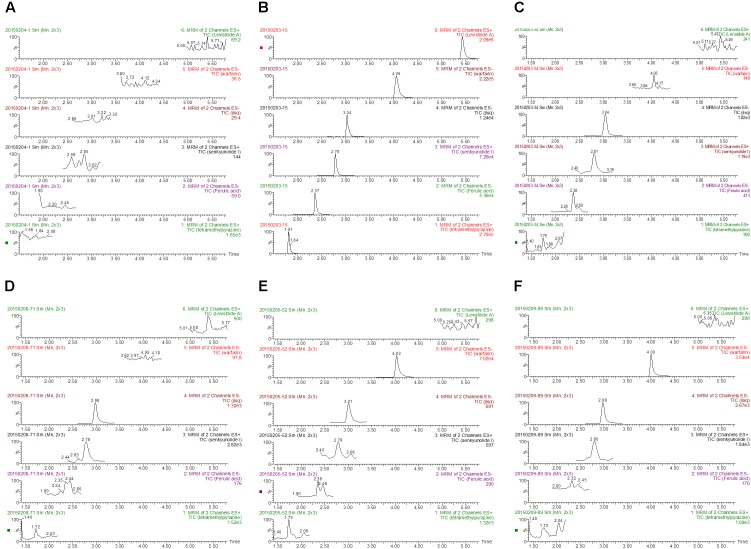
Representative MRM chromatograms of levistilide A, tetramethylpyrazine, senkyunolide I, ferulic acid, warfarin, and methyclothiazide in rats: **(A)** Blank plasma samples from healthy rats; **(B)** blank plasma samples spiked with levistilide A, warfarin, methyclothiazide, senkyunolide I, ferulic acid and tetramethylpyrazine; **(C)** plasma from healthy rats after a single administration of Chuanxiong; **(D)** plasma from rats with biliary drainage after a single administration of Chuanxiong; **(E)** plasma from healthy rats after the administration of warfarin and Chuanxiong; **(F)** plasma from rats with biliary drainage after the administration of warfarin and Chuanxiong (from the top to the bottom: levistilide A, warfarin, methyclothiazide, senkyunolide I, ferulic acid, and tetramethylpyrazine).

**FIGURE 2:**
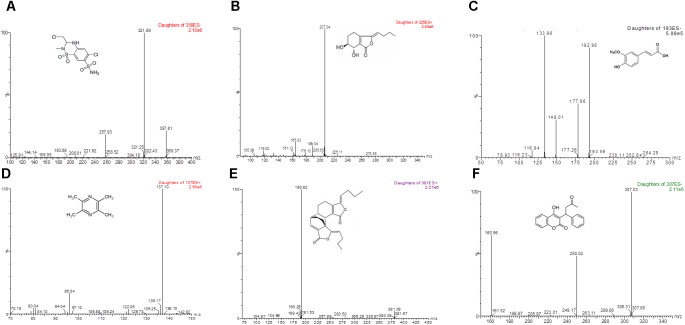
LC-MS/MS spectra of methyclothiazide, four marker compounds of Chuanxiong and warfarin. Methyclothiazide, warfarin, and ferulic acid can be ionized under negative ionization conditions, levistilide A, senkyunolide I, and tetramethylpyrazine ionized under positive ionization conditions. The most sensitive mass transitions for methyclothiazide [**(A)** the internal standard] were 357.81→321.89. for senkyunolide I **(B)** were 225.11→207.04, for ferulic acid **(C)** were 192.98→133.96, for tetramethylpyrazine **(D)** were 137.10→96.04, for levistilide A **(E)** were 381.09→190.92 and for warfarin **(F)**, were *m*/*z* 307.03→250.02.

The method exhibited good specificity, and no interference was found at the retention times of either IS or main bioactive components of Chuanxiong in plasma samples, which were used for the analysis. Typical MRM chromatograms were shown in Figure 1, and the UPLC-MS/MS spectra of the main bioactive components of Chuanxiong, IS and warfarin were displayed in Figure 2.

### Linearity and LLOQ of SEI

The linear regression equation of SEI was *y* = 0.00511*x-*0.0396 (*r* = 0.998852), the linearity range was 10–1,000 ng/mL, and the LLOQ was 5 ng/mL, where *x* referred to the SEI concentration (ng/mL), *y* indicated the ratio of the peak area of SEI to that of IS, and *r* was the correlation coefficient of the equation.

### Precision and Accuracy

The method presented good precision and accuracy with good inter-day and intra-day precision. All of the results were showed to be within the accepted variable limits, as demonstrated in Table 1.

**Table 1:** The intra-day and inter-day precision of senkyunolide I in rat plasma samples.

	Spiked concentration	Intra-day		Inter-day	
	(ng/ml)	Measured (ng/ml)	RSD%	Measured (ng/ml)	RSD%
	10	9.73 ± 0.46	4.73	10.32 ± 0.31	3.00
Senkyunolide I	100	103.76 ± 6.50	6.26	97.66 ± 4.70	4.81
	1000	1013.02 ± 73.2	7.23	1021.78 ± 67.4	6.60

### Pharmacokinetics Study

**FIGURE 3:**
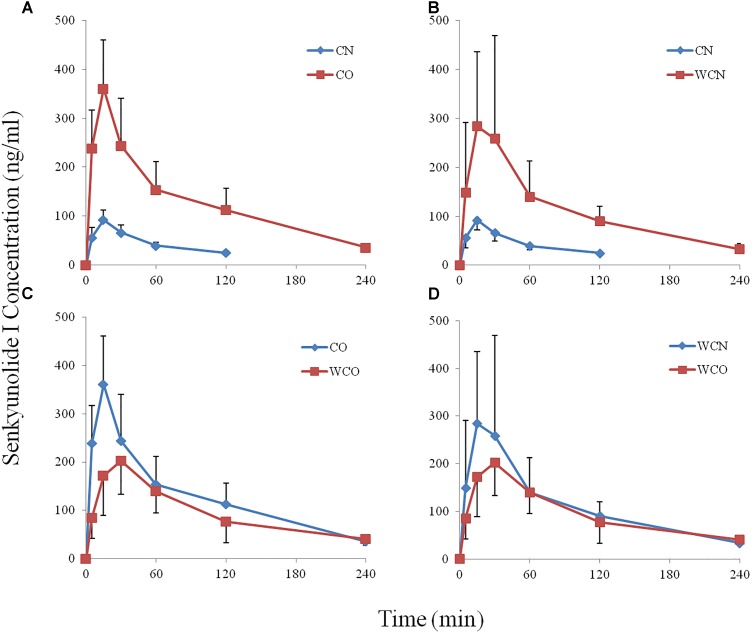
The effects of warfarin on Chuanxiong (Senkyunolide I) pharmacokinetics in rats (the mean plasma ± SD concentration-time curves for senkyunolide I, *n* = 8). **(A)** CO group compares to CN group; **(B)** WCN group compares to CN group; **(C)** WCO group compares to CO group; **(D)** WCO group compares to WCN group. Warfarin and biliary drainage both increase the bioavailability of Chuanxiong (Senkyunolide I) in rats. CN, healthy rats after a single administration of Chuanxiong; CO, rats with biliary drainage after a single administration of Chuanxiong; WCN, healthy rats after the administration of warfarin and Chuanxiong; WCO, rats with biliary drainage after the administration of warfarin and Chuanxiong.

Figure 3 showed the concentration-time curves for plasma SEI in the four groups (*n* = 8). The pharmacokinetics parameters (Table 2) of SEI were calculated by the DAS 2.1.1 software in non compartmental modeling.

**Table 2:** The comparison of senkyunolide I pharmacokinetics parameters (mean ± SD, *n* = 8).

Group	AUC_(0−t)_ (ng/mL⋅min)	MRT_(0−t)_ (min)	t_1/2z_ (min)	T_max_ (min)	*C*_max_ (ng/mL)
CN	5′564.63 ± 967.62	46.72 ± 2.44	62.01 ± 21.07	15.00 ± 0.00	92.33 ± 19.69
CO	30′376.22 ± 10′640.44^∗∗^	72.21 ± 13.95^∗∗^	70.54 ± 27.01	16.88 ± 5.30	371.49 ± 94.10^∗∗^
WCN	26′923.09 ± 12′611.33^∗∗^	80.84 ± 11.66^∗∗^	86.97 ± 20.69^∗^	16.88 ± 5.30	322.36 ± 213.54^∗∗^
WCO	21′539.44 ± 9′782.28	75.87 ± 16.42	85.92 ± 21.03	28.13 ± 5.30^## 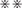 ^	208.85 ± 80.64^ 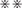 ^

*^∗∗^Compared to the CN group p < 0.01, ^∗^compared to the CN group p < 0.05; ^##^compared to the WCN group p < 0.01; ^
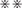
^compared to the CO group p < 0.01. CN, Healthy rats after a single administration of Rhizoma Chuanxiong; CO, rats with biliary drainage after a single administration of Rhizoma Chuanxiong; WCN, healthy rats after the administration of warfarin and Rhizoma Chuanxiong; WCO, rats with biliary drainage after the administration of warfarin and Rhizoma Chuanxiong.*

#### The Pharmacokinetics Parameter Comparisons of CO and CN Group

Table 2 indicated that there were significant differences in the pharmacokinetics parameters of SEI between the CO and CN groups. Compared with the CN group, the AUC_0−t_ and C_max_ of SEI in the CO group raised 5.46 and 4 times, respectively. The MRT_0−t_ was also delayed by 54.5% (*P* < 0.05).

#### The Pharmacokinetics Parameter Comparisons of WCN and CN Group

Further, Table 2 revealed that there were significant differences in the pharmacokinetics parameters of SEI between the CN and WCN groups. Compared with the CN group, the AUC_0−t_ and C_max_ of SEI in the WCN group increased to 4.84 and 3.5 times, respectively. At the same time, the MRT_0−t_ and t_1/2z_ in the WCN group were significantly increased (*P* < 0.05).

### The Pharmacokinetics Parameter Comparisons of WCO and CO Group

As shown in Table 2, compare to the CO groups, the C_max_ of the WCO group was remarkably decreased 43.78% (*P* < 0.01), the T_max_ of the WCO group delayed 66.6% (*P* < 0.01).

#### The Pharmacokinetics Parameter Comparisons of WCO and WCN Group

Compared with the CN group, the T_max_ of the WCO group was significantly delayed 66.6% (*P* < 0.01), there were non-statistical decreases in AUC_0−t_ and C_max_(*P* > 0.05).

## Discussion

It is generally acknowledged that among one hundred or more components of Chuanxiong, ligustilide is the most abundant compound and is the key bioactive ingredient (Yan et al., 2005). SEI is the main metabolites of ligustilide *in vivo* and *in vitro* (Tang et al., 2009; Zuo et al., 2011). However, ligustilide is unstable *in vivo* and *in vitro* (Yan et al., 2007), with poor oral bioavailability (approximately 2.6%) (Yan et al., 2008). Some scholars had confirmed that SEI is the richest ingredient and almost 4 times more than ligustilide in the water extraction of Chuanxiong (Yang et al., 2012). Our present research demonstrated that only SEI displayed detectable and complete concentration-time profile after oral administration water extraction of Chuanxiong. We did not observe the complete concentration-time profile of the other four main bioactive components. According to our previous study, the water extraction of Chuanxiong significantly affected the pharmacokinetics of warfarin in rat plasma (Li et al., 2016b), maybe SEI is the major bioactive constituent, further study on this problem should be performed.

The notable changes in AUC_0−t_ and C_max_ between the CO and CN group caused by biliary drainage indicates that the operation increased the bioavailability of SEI after oral administration of Chuanxiong. The results suggests that the biliary drainage operation might lead the absorption of SEI to be enhanced, just as the same to some literature materials has been reported, the operation improves the absorption of the representative active ingredients of Eucommiae cortex, such as geniposide and di-O-b-d-glucopyranoside (An et al., 2016). These phenomena could be preferably explained that the bile acids alternations, particularly in the liver and ileum. As the ligands of farnesoid X receptor and pregnane X receptor, bile acids play a regulatory role in affecting associated enzymes CYP3A11 and transporters Na^+^-taurocholate cotransporting polypeptide, bile salt export pump and sodium-dependent bile acids transporter at transcriptional level (Zhang et al., 2017). The enterohepatic bile circulation must have been broken by the biliary drainage operation in the present study, subsequently, altering related enzymes and transporters would affect the drug process *in vivo*. The major proposed metabolic pathways of SEI *in vivo* are glucuronidation, hydrolysis, and glutathione conjugation (Ma et al., 2016; Zhang et al., 2017). The high extent of glucuronidation indicates the possible presence of enterohepatic circulation, which was confirmed in the linked-rat model (Xing et al., 2005). The drug glucuronidation metabolite occurred in liver could be readily hydrolyzed by the intestinal microflora to free aglycone. SEI is rapidly absorbed into the intestine and 87% of that excreted by bile in normal intact rat (Ma et al., 2015). Owing to the broken enterohepatic circulation and the alternations of enzymes and transporters, the more drug in liver might get into the blood, and the less into the bile.

In normal rats, warfarin affects the pharmacokinetics of SEI according to the comparison of the WCN and CN group. Warfarin can exert anticoagulation after oral administration, and SEI significantly improves the plasticity and deformability of erythrocyte effectively (Hong et al., 2003), the absorption and distribution of drug tend to be strengthened due to their pharmacodynamic drug-drug interactions. This tendency is supported by our previous study; Chuanxiong significantly increases the bioavailability of warfarin in rat plasma (Li et al., 2016b). Similarly, the present data displayed that warfarin greatly increases the bioavailability of SEI after oral administration of Chuanxiong. Warfarin is a drug with high protein binding and some biologically active compounds of Chuanxiong can compete its serum albumin binding sites (Rimac et al., 2017), this might increase the concentrations of SEI and warfarin in rat plasma.

There is an opposite tendency in AUC and C_max_ of SEI between normal rats and rats subjected to biliary drainage co-administered orally warfarin and Chuanxiong. As shown in Table 2 and Figure 3, compared to the CO groups, the C_max_ of the WCO group is significantly decreased by 43.78%, but compared with the CN group, there is an obvious increase tendency in AUC and C_max_ of the WCN group. On one hand, the opposite tendency of pharmacokinetics effects might due to the broken of enterohepatic circulation. Our previous research had verified that warfarin existed enterohepatic circulation in rat after oral administration (Li et al., 2016a,b). We may speculate that warfarin influences the disposition of SEI by affecting its enterohepatic circulation process. In normal health rats, warfarin might compete the same enzymes and transporters with Chuanxiong in liver, the less of drug in liver might get into the bile, and the more into the blood. The similar competition would occur mainly in intestinal tract in rats with biliary drainage, and this competition might decrease the absorption of SEI. On the other hand, the biliary drainage operation would take some complex factors into the disposition process, and we further presume that whether diseases conditions produce the similar changes just displaying as in the present study. These corollaries will provide a chance to inquiry the absorption, distribution, metabolism, and elimination mechanism of SEI after oral administration of Chuanxiong in rats.

Although we displayed that warfarin impacted the pharmacokinetics of SEI in rats after oral administration Chuanxiong, it was not clear how it exerted its effects. The warfarin metabolism conjugation with glucuronic acid and/or sulfate, which takes place mainly in the liver, is the principal pathway. The detailed quantitative assessment and identification of the importance of various transporter/enzyme pathways in the disposition process of Chuanxiong *in vivo* are still deficient, although previous studies on its pharmacokinetics show that these processes might contain cytochrome P450s (CYPs), UDP-glucuronosyltransferases, sulfotransferases and monocarboxylic acid transporters (Poquet et al., 2008). It is believed that pharmacokinetics interaction between warfarin and Chuanxiong *in vivo* refers to several factors rather than above indicators.

After co-administration of warfarin and Chuanxiong, the biliary drainage operation significantly influenced the disposition of SEI in rats. Moreover, the drug warfarin significantly affected the disposition of the main herbal component SEI, partly due to the enterohepatic circulation of warfarin. The present study integrated with our published researches indicates that it is complicated in the interactions of herbal and Western medicine based on pharmacokinetics. This study provides an urgent evidence for drug-herb interactions.

## Author Contributions

HLi, TT, and YW did conception and design. HLi, YJ, YW, and JT carried out the experiments. HLv and CG acquired the data. GY and HX analyzed and interpreted the data. HLi drafted the manuscript. All authors critically revised the manuscript.

## Conflict of Interest Statement

The authors declare that the research was conducted in the absence of any commercial or financial relationships that could be construed as a potential conflict of interest.
